# Sodium pyruvate ameliorates cognitive dysfunction by expanding hippocampal endogenous neural stem cells in tBCCAO mice

**DOI:** 10.3389/fcell.2026.1782699

**Published:** 2026-03-11

**Authors:** Jia He, Lici Yang, Zican Wang, Wenwen Liu, Benjun Qi, Pengyue Li, Yongwei Pan, Yongjian Jiang, Dongyu Ding, Ge Yan, Zijia Liu, Lili Yuan, Yang Gao

**Affiliations:** 1 College of Basic Medicine, Jining Medical University, Jining, Shandong, China; 2 College of Clinical Medicine, Jining Medical University, Jining, Shandong, China; 3 College of Integrative Medicine, Jining Medical University, Jining, Shandong, China

**Keywords:** endogenous neural stem cells, H3K9ac, hippocampal, P300, sodium pyruvate

## Abstract

**Background:**

After a stroke, many survivors experience post-stroke cognitive impairment (PSCI), a frequent clinical problem that might continue for an extended timeframe. Nerve regeneration is a crucial aspect of the body’s self-repair mechanism following a stroke. While Sodium Pyruvate (SP) exhibits notable neuroprotective properties, its potential role in facilitating nerve regeneration requires further investigation.

**Objective:**

Thorough investigation into how cerebral ischemia-reperfusion injury is repaired and the function of SP in healing ischemic stroke damage.

**Methods:**

A temporary bilateral common carotid artery occlusion model (tBCCAO) was used to induce cerebral ischemic injury in mice. Laser scattering technique used to evaluate blood flow variations in the mouse brain. The water maze served as a tool to measure the learning and memory capabilities of the mice. The expansion of neural stem cells (NSCs) pool were evaluated through immunofluorescence, Western blot, and qPCR assays.

**Result:**

SP significantly mitigates pathological brain tissue damage, enhances learning and memory in mice, stimulates NSC pool expansionin the hippocampal subgranular zone (SGZ) region, and upregulates the expression of SOX2, p300, H3K9ac, and DCX proteins *in vivo*.

**Conclusion:**

According to our findings, the mechanisms by which SP enhances the growth of internal NSCs and the maturation of immature neurons may involve the p300-H3K9ac-DCX signaling pathway, suggesting a new therapeutic target for stroke recovery.

## Introduction

Post-Stroke Cognitive Impairment (PSCI) describes the range of cognitive decline that can occur following a stroke, from minor issues to severe dementia. The total prevalence of PSCI changes from 20% to 80% (Zhao et al., 2024). Patients with PSCI may have deficits in several cognitive domains such as attention, computation, delayed recall, memory, executive functioning, and visuospatial abilities (Siow et al., 2024; Aam et al., 2021). This is closely related to cerebral ischemia-reperfusion injury (CI/RI). CI/RI’s pathological mechanism is complicated, involving known processes like inflammatory response, autophagy, mitochondrial dysfunction, and calcium overload (Wei et al., 2022). The impact of CI/RI includes damage to neuronal and glial cells and tissue disruption, resulting in neurological dysfunction. Although neurons are harmed, they are not deceased. They can mend themselves, and one way they do this is by differentiating and regenerating neural stem cells (Tang et al., 2023).

The focus on neurogenesis after a stroke has grown in recent decades, presenting a promising therapeutic option for ischemic stroke. These endogenous NSCs can initiate adult neurogenesis to replace neurons lost after an ischemic stroke, which involves their growth and differentiation (Tang et al., 2023). Histone acetylation, which is an essential epigenetic alteration associated with gene transcription, is believed to have a significant impact on the differentiation of NSCs (Fukuda et al., 2024). The lysine acetyltransferases CBP and its paralogue p300 play roles in transcriptional coactivation, neural plasticity, and memory. p300/CBP is considered to be involved in the differentiation and maturation of adult neuronal stem cells, producing multi-branched newborn neurons with double cortical (DCX)-positive function in the hippocampus’s subgranular zone (SGZ) (Smitha et al., 2024).

Recent studies have indicated that sodium pyruvate (SP) provides neuroprotection in multiple brain injury models (Moro and Sutton, 2010; Moro et al., 2016; Wang et al., 2009). Those investigations have demonstrated that SP protects the brain of a neonate from injury caused by hypoxia and ischemia through the conservation of brain metabolic and mitochondrial functions (Moro et al., 2016).

Is it possible for SP to stimulate NSC proliferation and differentiation through p300 activation to mitigate PSCI? This research examines the growth and specialization of NSCs through *in vivo* experiments to confirm its effects. We intended to study the CI/RI repair mechanism by establishing an *in vivo* tBCCAO mouse model and to elucidate the SP repair mechanism in PSCI. The purpose is to lay down the pharmacological groundwork for the clinical treatment of PSCI.

## Materials and methods

### Experimental animals

Shandong Jinan Pengyue Laboratory Animal Breeding Co. supplied 8- to 10-week-old male C57BL/6J mice, each weighing 18–20 g. The Ethics Committee at Jining Medical University in China gave their approval for the animal experiments (JNMC-2021-DW-019). Mice experienced a 12-h alternation of light and dark, with environmental conditions of 22 °C–25 °C and 52%–56% humidity. A week of standard food and water was given to all the mice after adaptive feeding.

### Reagents

Sodium pyruvate (SP) was obtained from Yuanye Biotechnology Co. (Shanghai, China). p300(Cell Signaling Technology, 86377), sox2 (ab0224), BDNF (ab108319), DCX (ab18723), H3(Cell Signaling Technology, 4499) and H3k9ac (Cell Signaling Technology,9649) antibodies were acquired from Jining Qiancunxin Biotechnology Co., Ltd. Instrument PeriCam PSI HR (Perimed, Ard more, PA), Morris Water maze (Techman instrument Co.,Ltd., Sichuan, China).

### tBCCAO model building

To induce PSCI, the tBCCAO surgery was executed following a technique that has been described before (Kita et al., 2025). The mice were put under anesthesia using 1.25% tribromoethanol. Microvascular clips were used to occlude both the right and left common carotid arteries (CCAs) for 20 min after they were isolated from nearby nerves and sheaths. Following this, the clips were taken away to facilitate CBF reperfusion, and the incisions were stitched. In the control group, mice were subjected to a 20-min sham operation without CCA occlusion. Afterward, they were housed with unrestricted availability of food and water.

### Experimental group information

Mice were allocated into three groups randomly: the Sham surgery group, the tBCCAO group, and the SP group (1, 2 g/kg), which was a refinement of the dosage used in the existing studies (Moro and Sutton, 2010; Tang, 2019). Mice underwent intragastric administration for 14 days, with Samples were obtained 2 h after the last dose was given on the last day.

### Hematoxylin-eosin (HE) staining

The brain tissues of mice were harvested after cardiac perfusion and preserved in a fixative with 4% paraformaldehyde. The fixed brain tissue was dehydrated using graded alcohol, cleared with xylene, and then submerged in liquid paraffin. After completing paraffin infiltration, The tissue underwent cooling, sectioned into 5 μm pieces, placed on slides, then allowed to dry. Xylene was employed for dewaxing, after which the samples were hydrated using alcohols of descending concentrations and then immersed in distilled water for staining. Following staining, the sections were dehydrated using alcohols of varying concentrations, purified with xylene, enveloped in neutral gum, and enveloped in glass slides for examination under a microscope.

### Nissl staining

The sections were immersed in ethanol solutions of 100%, 90%, 70%, and 50% for 5 min each, followed by a water wash, and stained with 0.1% cresyl acetate violet at 37 °C for 10 min. After washing with water, the sections were soaked in 50% ethanol and 70% ethanol for 5 min each, then in 95% ethanol for 1 min, and finally in 100% ethanol twice for 5 min each. Following this, the tissue was dipped in xylene and covered using DPX.

### Neurological-deficit score (NDS)

Standard procedures were used to perform neurological-deficit scoring to evaluate the level of impairment after surgical interventions (Kushwaha et al., 2025). The evaluation relied on the presence and intensity of certain behavioral symptoms. In short, 0 points were awarded if no symptoms or disorders were found. Award 1 point if unilateral or bilateral ptosis is observed. 1.5 points were given for the observation of body flexion, and 2 points were given for the presence of both forelimb tremor and body flexion. Give 2.5 points for motor weakness and 3 points for the presence of all symptoms at the same time.

### Morris water maze

Mice participated in a four-day water maze experiment, where their escape latency and learning performance were evaluated over the first 3 days. Memory was evaluated on the 4th day by determining how long they stayed in the original platform quadrant and how often they crossed the platform areas.

### Immunofluorescence

The brain tissue was preserved in 4% paraformaldehyde, treated with 30% sucrose for dehydration, and then cryosectioned. Using Triton X-100, sections were permeabilized, held at room temperature for 30 min and then three rinses with PBS were performed. Blocking was done for thirty minutes at room temperature with 5% BSA. The primary antibody DCX (ab18723, abcam), SOX2 (ab0224, abcam), and P300 (86377, Cell Signaling Technology) were readied and incubated overnight at a temperature of 4 °C. The application of fluorescent secondary antibodies was followed by incubation of the cells in the dark at ambient temperature. The sample was rinsed three times using PBS, covered with DAPI stain, and then shield from light for 5 min at ambient temperature. Using a fluorescence microscope, the sealed sections were imaged.

### qRT-PCR assays

Obtaining total RNA from tissue through the VeZol-Pure Total RNA Isolation Kit (Vazyme #RC202). Following the instructions, 1 μg of purified RNA was synthesized using the Evo M-MLV One-Step RT-qPCR Kit (Accurate Biology). qRT-PCR was conducted using a real-time PCR machine (Roche). The qRT-PCR assay results represent the mean values from two independent RNA extractions. The cycling program for PCR started with 2 min at 94 °C, then 40 cycles of 94 °C for 15 s, 60 °C for 20 s, and 72 °C for 40 s. The comparative threshold cycle (Ct) method was applied for data analysis.

### Western blot

The brains were promptly removed, and the hippocampus tissues were separated, then 1 mL of RIPA lysate with PMSF was added for complete grinding. To collect the supernatant, samples are left on ice for a duration of 30 min and then centrifuge at 4 °C for 5 min at 12,000 rpm/min. A 4:1 mixture of total protein from each group and 5x sample buffer was boiled for 10 min. Gels for SDS-PAGE separation and concentration were prepared with suitable concentrations, based on the protein’s molecular weight. Following the addition of samples, electrophoresis was carried out, and the samples were wet transferred to a PVDF membrane. The membrane was blocked with milk for an hour and then incubated overnight at 4 °C with the specific primary antibody. Following three 10-min washes with TBST, the membrane was treated with the corresponding secondary antibody and left to incubate for 1 h at room temperature. The membrane made of PVDF underwent three further 10-min washes with TBST, after which the developer reagent was applied to specific spots and exposed in a gel imager.

### Statistical analysis

SPSS 23.0 software was employed to process the experimental results, which were expressed as mean ± standard deviation. Group differences were analyzed using Student’s t-test, one-way ANOVA or two-way repeated measures ANOVA, with a p-value below 0.05 indicating significance.

## Results

### SP provides a protective effect on the brains of tBCCAO mice

**FIGURE 1:**
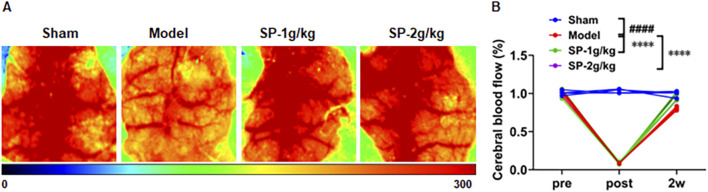
Effect of SP on cerebral blood flow recovery in tBCCAO mice. **(A)** Results of laser speckle cerebral blood flow imaging (n = 4). **(B)** Summary of cortical CBF data in the ROI 5 min before tBCCAO, 20 min following CCAs occlusion, and after 2 weeks of treatment. ####p < 0.0001 vs. Sham, ****p < 0.0001 vs. Model.

**FIGURE 2:**
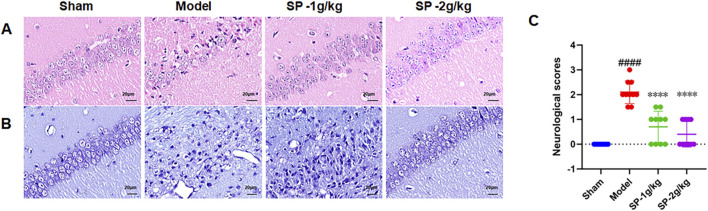
Effects of SP on Neuronal Repair in the DG region of tBCCAO Mice. **(A)** Hematoxylin and Eosin (HE) were used to stain neuronal cells in the hippocampal DG area of mice after undergoing tBCCAO and their respective treatments. **(B)** Nucleic acid staining (Nissle)of hippocampal neurons in tBCCAO mice administered with SP. **(C)** Neurologic deficit scores (NDS) across different groups (n = 10). ####p < 0.0001 vs. Sham, ****p < 0.0001 vs. Model.

To confirm the creation of the tBCCAO model in mice, we monitored cerebral blood flow (Figures 1A,B). Following the modeling process, the mice were assigned to groups at random, There was a notable reduction in cerebral blood flow within the model group. Recovery pattern of cerebral blood flow post SP treatment. Pathological injury and neuron cells were significantly elevated after modeling, as shown by HE and Nissle staining (Figures 2A,B). SP reduced brain tissue damage, brain cell apoptosis, and improved neurological scores (Figure 2C). These findings propose that sodium pyruvate could mitigate damage to brain tissue following CI/RI.

### SP regulates activation of NSCs in the hippocampus of tBCCAO brains

**FIGURE 3:**
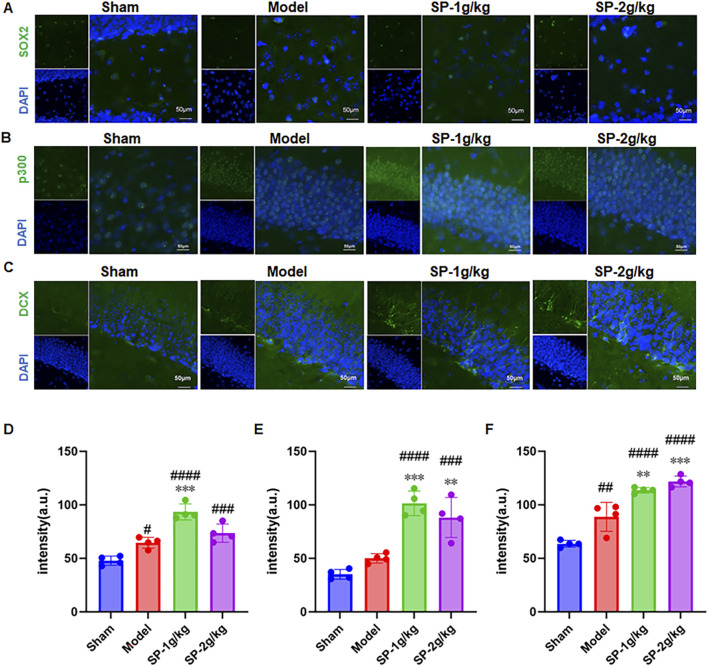
Effects of SP Treatment on Proliferation and Differentiation of Neural Stem Cells in the Dentate Gyrus of tBCCAO Mice. **(A–C)** Immunofluorescence detection of SOX2, p300, and DCX expression (Magnification×400,n = 4) **(E,F)**. Assessment of the graph’s results through quantitative analysis. #p < 0.05 vs. Sham, ##p < 0.01 vs. Sham, ###p < 0.005 vs. Sham, ####p < 0.0001 vs. Sham, **p < 0.01 vs. Model, ***p < 0.001 vs. Model.

**FIGURE 4:**
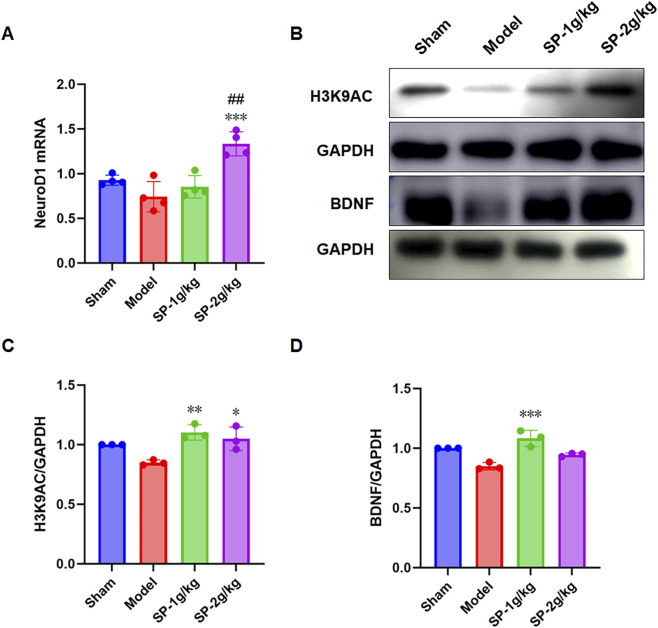
Effects of SP Treatment on Hippocampal H3K9ac and BDNF Protein and NeuroD1 mRNA Expression in tBCCAO Mice. **(A)** Changes in NeuroD1 mRNA Expression in the Hippocampus of Mice Across Groups (n = 4). **(B)** Changes in H3K9AC and BDNF protein expression in the hippocampus of mice across different groups (n = 3). **(C,D)** Quantitative analysis of the result graph ##p < 0.01 vs. Sham, *p < 0.05 vs. Model, **p < 0.01 vs. Model.

NSCs could both self-renew and transform into glial cells and neurons. SOX2 has been a core transcription factor in neural stem cell fate determination (Rojo et al., 2022). NeuroD1, alternatively known as BETA2, is a transcription factor classified under the basic helix-loop-helix (bHLH) type that connects with E-box sequences on DNA and enlists histone acetyltransferases, such as p300, to promote chromatin relaxation, thereby initiating the expression of neural genes (Pataskar et al., 2016). To explore the capabilities of SP in aiding NSCs to become neurons and mitigate effects, we used immunofluorescence for SOX2 on the 77th day and qPCR for NeuroD1 on the 14th day. According to the results, there was a significant increase in SOX2+ cells in the hippocampal DG area (Figures 3A,D)and in the mRNA level of NeuroD1 in the hippocampus for the SP treatment group (Figure 4A). This discovery indicated that SP might trigger NSCs to begin differentiating.

### SP expanded the pool of neural stem cells may closely relate to P300/H3K9ac in the hippocampal SGZ region of tBCCAO brains

P300 served as a crucial transcriptional co-activator for NeuroD1. The interaction between these two proteins occurs directly at the C-terminus, facilitating the incorporation of p300’s histone acetyltransferase activity into the promoters of NeuroD1 target genes (Van de Velde et al., 2019). The formation of activated chromatin modifications through this process significantly stimulates the expression of genes pertinent to neural differentiation. To determine whether the molecular process by which SP facilitates endogenous NSCs differentiation involves p300, the immunofluorescence triple staining was utilized to assess the expression of p300 (Figures 3B,E). The results indicated that the fluorescence intensities of p300 increased after SP treatment.

The primary acetyltransferase, P300, was responsible for acetylating histone H3 at lysine 9 (H3K9), which was essential for gene activation related to neurogenesis in hippocampal NSCs through H3 upregulation (Zhou et al., 2019). Enhanced p300 activity resulted in a marked rise in H3K9 acetylation, which supports the expansion and maturation of NSCs and enhances neuroplasticity in the adult hippocampus (Zhou et al., 2019). The protein level of H3K9AC in SP-treated groups post-tBCCAO was further investigated using WB analysis on the 14th day after SP treatment. In the SP group, H3K9ac protein levels were greater than those in the tBCCAO group (Figures 4B,C).

Subsequent immunofluorescence investigations into the expression of DCX, a gene implicated in the maturation of immature neurons within the DG of the hippocampus (Yoo et al., 2011), demonstrated a notable enhancement in the population of DCX + neurons in this specific region following 14 days of treatment with SP (Figures 3C,F). The survival, growth, and differentiation of newly created neurons are maintained and regulated by BDNF (Charou et al., 2024). The assessment of BDNF protein expression was performed via Western blot (Figures 4B,D). Compared to the tBCCAO group, the SP group had greater BDNF protein levels.

Our data indicated that the p300/H3K9ac pathway may contribute to the cognitive improvements.

### SP ameliorated cognitive impairment in tBCCAO mice

**FIGURE 5:**
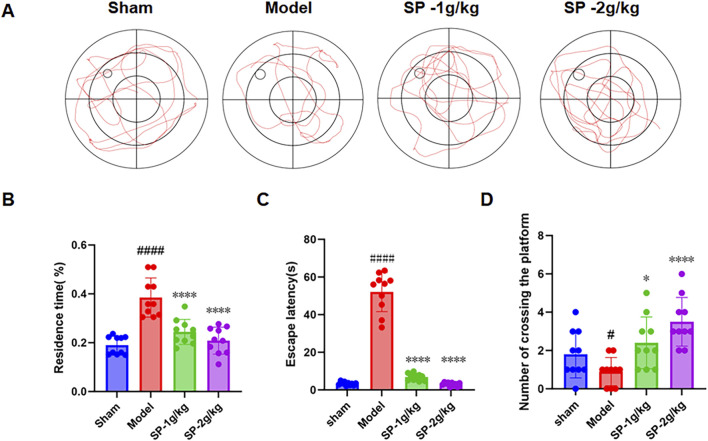
Influence of SP on Mental Function in tBCCAO Mice. **(A)** Morris Water Maze Trajectory Diagram (n = 10). **(B–D)** Quantitative analysis of the result graph. #p < 0.05 vs. Sham, ####p < 0.0001 vs. Sham, *p < 0.05 vs. Model, ****p < 0.0001 vs. Model.

After a 14-day SP treatment, the MWM test was employed to evaluate the learning and memory skills of tBCCAO mice. There were marked differences in spatial learning among the three groups, as evidenced by the time taken to discover the escape platform in the water maze (Figures 5A,B). The tBCCAO mice took significantly longer to escape compared to the Sham group, while the SP treatment notably decreased the delay, highlighting better cognitive outcomes in the SP group (Figure 5C). The probe trial was conducted to assess memory retention after a 24-h dela. In the tBCCAO group, platform crossings were significantly fewer than in the Sham group, but the SP group showed a marked improvement (Figure 5D). Altogether, the study results demonstrated that SP significantly enhanced the cognitive functions related to learning and memory in mice after tBCCAO, which could be connected to the promotion of NSC pool expansion and neuron survival by SP.

## Discussion

Our research has indicated that SP treatment reduces neurological impairments and improves cognitive deficits in tBCCAO mice. We have presented novel findings in this study, showing that SP promotes neurorestoration by encouraging NSC proliferation in a living system. The potential molecular mechanism by which SP enhances neurogenic potential is by acting as a p300 activator, increasing H3K9 acetylase activity, which in turn causes an increase in the expression of DCX, thereby inducing the development and specialization of endogenous NSCs, and facilitating the restoration of cognitive function after stroke.

Some people who suffer from an ischemic stroke may naturally recover to some extent, even without medical care (Liu et al., 2023). Recent findings suggest that this result can be attributed to endogenous NSCs residing in the adult subventricular zone (SVZ) and SGZ (Jin and Galvan, 2007). Yet, the efficiency of neurogenesis in repairing neural tissue after an ischemic stroke is still low. Drug therapies or transdifferentiation strategies might offer additional solutions to address this inefficiency. Furthermore, *in vivo* tracking of neurogenesis will definitely assist clinical research in the treatment of ischemic stroke patients. Earlier research has shown that PS had therapeutic benefits on cerebral ischemia, but its impact on transient cerebral ischemia and NSCs reperfusion injury activation was not documented.

SP has demonstrated substantial neuroprotective effects in various models of neural injury, including oxidative stress, ischemia, and trauma (Moro et al., 2016; Rong et al., 2015). The primary mechanisms underlying these effects encompass direct scavenging of hydrogen peroxide, provision of metabolic substrates, inhibition of NF-κB-mediated inflammation, reduction of zinc toxicity, and preservation of mitochondrial function (Tang, 2019).

Earlier studies have used rat models to explore the effects of SP at a top dose of 1 g/kg (Moro and Sutton, 2010; Tang, 2019), but its impact on tBCCAO mouse models remains unreported. In our research, tBCCAO mice received pyruvate in doses of 1 g/kg and 2 g/kg. There was a significant boost in cerebral blood flow in the SP-treated tBCCAO mice, most notably at the 2 g/kg dose. Moreover, histological analysis with HE and Nissl staining showed that SP significantly reduced pathological symptoms in tBCCAO mice, especially in the hippocampal DG region. This effect could be associated with the decrease in inflammation (Moro and Sutton, 2010). Accumulated evidence over the past forty years reveals that neural stem cells inhabit the subgranular zone of the hippocampal DG, where they produce GCNs throughout adulthood (Pfeiffer et al., 2018). Could SP potentially activate these cells to help repair damage? Our study revealed that following SP treatment 7days, the number of SOX2-positive NSCs in the DG region significantly increased. Furthermore, the levels of neurod1 mRNA were markedly elevated. SOX2 is a transcription factor found in neural progenitor cells that also controls the nestin core enhancer and is essential for maintaining NSCs and brain development (Graham et al., 2003; Jin et al., 2009; Zhang and Cui, 2014). In undifferentiated NSCs, SOX2 forms a complex with histone deacetylase HDAC1, which binds to the Sox/LEF site on the *NeuroD1* promoter to downregulate NeuroD1 transcription (Chen et al., 2025). During the neuronal differentiation phase, Wnt/β-catenin signaling becomes active. An activating complex is created by β-catenin in place of SOX2/HDAC1, leading to elevated *NeuroD1* gene expression (Jiang et al., 2020). The activity of NeuroD1 as a transcription factor is highly dependent on its association with the coactivator p300 (Sharma et al., 1999). Acting as a histone acetyltransferase, p300 is attracted to the promoters of NeuroD1-associated genes, where it enhances the modification of histones H3 and H4 through acetylation and loosens chromatin structure (Van de Velde et al., 2019). This significantly enhances the transcriptional activity of NeuroD1 target genes, including Insulin, Neurogenin, BDNF, DCX, and others (Ray et al., 2014). In this research, administering sodium pyruvate for 14 days notably elevated p300 expression in the hippocampal DG area. At the same time, there were significant rises in H3K9ac and BDNF protein levels, reinforcing SP’s activity. While the correlation between p300 upregulation, increased H3K9 acetylation, and elevated DCX expression is compelling and provides a strong foundation for our hypothesis, it does not prove causality. Our definitive proof would require intervention experiments, such as pharmacological inhibition or genetic knockdown of p300, to determine if the SP-induced upregulation of DCX and subsequent behavioral effects are directly dependent on this pathway.

Our research offers preliminary evidence supporting the idea that SP might aid cognitive recovery partly by influencing neural progenitors. The research indicates a link between SP treatment, epigenetic modifications, and neurogenic markers, providing a basis for additional mechanistic studies. Yet, there are limitations to using SOX2 and DCX as exclusive markers. Verifying cell proliferation and tracking the survival and integration of new neurons in adult neurogenesis would require additional techniques, such as using thymidine analog labeling like BrdU or EdU. Sodium pyruvate is known for its antioxidant properties, which can directly shield hippocampal neurons from ischemic harm. Its possible anti-inflammatory properties might maintain the neurogenic environment and synaptic function without necessarily encouraging the creation of new neurons. The potential that improved neuronal survival and synaptic plasticity, rather than neurogenesis itself, are the main factors behind behavioral recovery, with the observed changes in NSCs being a subsequent or concurrent occurrence. A key limitation of this research is the insufficient experimental data to prove a causal relationship between the changes in NSC markers and the behavioral improvements. While the timing relationship is notable, we cannot ignore the possibility that SP’s cognitive benefits are mediated through other mechanisms, like direct neuroprotection or anti-inflammatory actions. To accurately assess the role of newly formed neurons in the recovery process after SP treatment, future studies will need to use strategies like pharmacological or genetic inhibition of adult neurogenesis.

## Data Availability

The datasets presented in this study can be found in online repositories. The names of the repository/repositories and accession number(s) can be found in the article/supplementary material.
